# Exploring the fatigue phenomenon in individuals with Parkinson’s disease: an Italian multicenter cross-sectional study

**DOI:** 10.1007/s13760-026-03039-3

**Published:** 2026-05-01

**Authors:** Elisabetta Sarasso, Elisa Pelosin, Ilaria Ruotolo, Manuela Maieron, Alessandro Ferrero, Francesca Quarone, Andrea Grassi, Martina Putzolu, Andrea Gardoni, Giovanna Lagravinese, Francesca Di Biasio, Federica Agosta, Giovanni Galeoto, Giovanni Fabbrini, Laura Avanzino, Massimo Filippi, Susanna Mezzarobba

**Affiliations:** 1 Neuroimaging Research Unit, Division of Neuroscience, IRCCS San Raffaele Scientific Institute, Milan, Italy; 2 Vita-Salute San Raffaele University, Milan, Italy; 3 Neurotech Hub, Vita-Salute San Raffaele University, Milan, Italy; 4 Department of Neuroscience, Rehabilitation, Ophthalmology, Genetics and Maternal Child Health, University of Genoa, Genoa, Italy; 5 IRCCS AOM Ospedale Policlinico San Martino, Genoa, Italy; 6 Department of Human Neurosciences, Sapienza University of Rome, Rome, Italy; 7 Istituto di Medicina Fisica e Riabilitazione, Azienda Sanitaria Friuli Centrale, Udine, Italy; 8 Azienda Sanitaria Locale Cuneo 1, Cuneo, Italy; 9 Ospedale Martini, Azienda sanitaria Locale Città di Torino, Torino, Italy; 10 Department of Experimental Medicine (DIMES), Section of Human Physiology, University of Genoa, Genoa, Italy; 11 Neurology Unit, IRCCS San Raffaele Scientific Institute, Milan, Italy; 12 Neurorehabilitation Unit, IRCCS San Raffaele Scientific Institute, Milan, Italy; 13 IRCSS Neuromed, Pozzilli, (IS) Italy; 14 Neurophysiology Service, IRCCS San Raffaele Scientific Institute, Milan, Italy

**Keywords:** Fatigue, Parkinson’s disease, Non-motor symptoms, Motor complications

## Abstract

**Background:**

Fatigue is a common and disabling non-motor symptom in Parkinson’s disease (PD), significantly affecting patients’ quality of life. However, it is often underdiagnosed due to its subjective nature and the lack of a clear definition, hindering the development of effective treatments.

**Objectives:**

This study aims to investigate the prevalence of fatigue and its associations with sociodemographic factors, disease severity, levodopa equivalent daily dosage (LEDD) and motor, non-motor, and cognitive symptoms in an Italian cohort of patients with PD.

**Methods:**

An observational cross-sectional study was carried out in three Italian centers from January to May 2024. One hundred PD patients (H&Y ≤ 4) were assessed using validated tools: Parkinson Fatigue Scale (PFS), Fatigue Severity Scale, and Modified Fatigue Impact Scale. Motor and non-motor signs and symptoms, cognitive status, and LEDD were analyzed using non-parametric tests and Spearman’s correlations. Fatigue prevalence was determined based on PFS score ≥ 3.09.

**Results:**

Fatigue was present in 36% of patients, more prevalent in women and more severe in those with H&Y > 2. Fatigue correlated strongly with non-motor symptoms (MDS-UPDRS Part I; ρ > 0.6, *p* < 0.001) and moderately with motor complications (0.4 < ρ < 0.5, *p* < 0.001), but weakly with disease duration, LEDD and age (ρ < 0.3, 0.002 < *p* < 0.05). Significant intercorrelations among fatigue scales supported their ability to consistently measure the fatigue construct.

**Conclusions:**

Fatigue in PD is a multidimensional phenomenon influenced mainly by non-motor symptoms. Gender-specific differences and the association with disease progression underscore the need of comprehensive and integrated management strategies to address this challenging symptom.

## Introduction

Parkinson’s disease (PD) is a progressive neurodegenerative disorder characterized predominantly by both motor and Non-Motor Symptoms (NMS) [1, 2]. Among NMS, fatigue, defined as a persistent and overwhelming sense of physical and/or mental exhaustion that is disproportionate to the level of exertion and not adequately alleviated by rest [3], is one of the most prevalent and debilitating, affecting approximately 33% to 58% of individuals with PD [4].

In the PD population, fatigue is more common among women [5–8], in individuals with postural instability and gait disorders phenotype [9, 10], and in patients with early cognitive decline [11, 12]. Fatigue appears to increase with the progression of PD severity [8], although it may also present in the premotor stage of the disease [13]. Moreover, fatigue should be distinguished from sleepiness, lack of motivation, or depression, even if these NMS are usually overlapped [8, 14, 15].

Despite its high prevalence, fatigue remains underdiagnosed, poorly understood, and often under prioritized in both research and clinical practice [3–6], partly due to its subjective nature and lack of standardized diagnostic criteria or assessment tools [12, 16, 17].

The pathophysiology of fatigue in PD is multifaceted, involving a dynamic interplay of neurochemical, structural, and psychosocial factors [18–26]. Neurobiological evidence implicates disruptions in dopaminergic, serotonergic, and noradrenergic pathways, which are critical for maintaining energy homeostasis and motivation [24, 25]. Structural and functional alterations in regions such as the prefrontal cortex, basal ganglia, and limbic system further contribute to the dysregulation of neural networks implicated in the perception of fatigue [18, 21, 23, 26]. Moreover, psychiatric comorbidities such as depression and anxiety, which are common in PD, often exacerbate fatigue, highlighting its biopsychosocial complexity. Additionally, fatigue significantly impacts functional independence, daily activities, and caregiver burden, presenting a major challenge for healthcare systems.

To date, the management of fatigue in PD remains suboptimal, with few evidence-based therapeutic options available. Pharmacological interventions, primarily dopaminergic therapies, show limited efficacy [27–29]. Non-pharmacological approaches, such as exercise programs, cognitive-behavioral therapy, and psychosocial interventions, show promising results but require further validation [28].

This study aims to provide a comprehensive analysis of fatigue in an Italian cohort of PD patients. By investigating fatigue prevalence and its associations with sociodemographic factors, disease severity, motor, non-motor and cognitive symptoms, this study should offer new insights to understand fatigue as a multidimensional phenomenon and to reason about clinical strategies for the effective management of such complex symptom.

## Methods

### Study design and population

An observational cross-sectional study was conducted at three centers (IRCCS San Raffaele Scientific Institute of Milan, University of Genoa, Sapienza University of Rome). The study was carried out in accordance with local ethical committee (EC) procedures (EC IRCCS San Raffaele Scientific Institute of Milan - protocol No. 68/int/2019; EC University of Genoa - protocol No. 2025/25; EC Sapienza University of Rome – Rif. 5830 protocol No 0428/2020) and the principles outlined in the Declaration of Helsinki concerning research involving human participants. Prior to participation, written informed consent was obtained from all participants.

Participants with idiopathic PD, diagnosed according to the London Brain Bank criteria [30], were consecutively enrolled during routine medical examination. Eligibility criteria included: Hoehn and Yahr (H&Y) score ≤ 4; stable antiparkinsonian medication for at least one month; Montreal Cognitive Assessment (MoCA) ≥ 17; and independent walking (with assistive devices if necessary). Exclusion criteria included: history of neurologic disorders other than PD; psychotic disorders and/or previous treatment with anti-psychotic drugs; and major depressive episodes or conditions (e.g. visual, orthopedic impairments) that could limit data collection.

### Data collection and management

Demographic data (age, sex, disease duration, educational level) and clinical data (H&Y stage, MDS-UPDRS score, MoCA score) were collected. Levodopa equivalent daily dosage (LEDD) was calculated according to standardized conversion factors [31].

Fatigue was assessed using three validated patient self-reporting scales: the Italian versions of Parkinson Fatigue Scale (PFS-16), Fatigue Severity Scale (FSS-9) [32, 33], and Modified Fatigue Impact Scale (MFIS) [34]. In the PFS-16, a cut-off score ≥ 3.09 points has been previously considered discriminatory for the presence of fatigue in Italian population (using the PFS-16 polytomous correction method) [32] and was used to define fatigue status in our sample, categorizing participants into “PD-fatigue” and “PD-non-fatigue” subgroups. Participants were further stratified into subgroups based on disease stage (H&Y < 2, H&Y = 2, H&Y > 2) and sex. All clinical evaluations were performed during the “on” medication state (i.e., one/two hours after the dopaminergic medication intake).

### Statistical analysis

Statistical analyses were conducted using IBM SPSS Statistics version 23. The normality of data distribution was assessed using the Shapiro-Wilk test, and sphericity was evaluated using the Mauchly’s test. Descriptive statistics (mean, standard deviation, and range) were used to characterize the whole sample and each subgroup. Between group comparison for continuous variables was assessed using non-parametric tests (Mann-Whitney, Kruskal-Wallis), while categorical variables were analyzed using Chi-square tests.

Spearman’s correlation coefficients (ρ) were used to examine associations between fatigue and clinical variables in the total sample of participants and in the fatigue subgroup.

Significance level was considered with a p value < 0.05 (all reported p-values are two-tailed). Effect sizes were classified as negligible (ρ < 0.1), weak (0.1 ≤ ρ < 0.3), moderate (0.3 ≤ ρ < 0.5), or strong (ρ ≥ 0.5) [35].

### Data availability

The dataset used and analyzed during the current study is available from the corresponding author upon request to qualified researchers (i.e., affiliated to a university or research institution/hospital).

## Results

### Characteristics of participants

The demographic and clinical characteristics of participants are reported in Table 1. A total of 100 PD patients (59 males; mean age 68.02 ± 8.40 years) were enrolled, 36 of whom exhibited fatigue (PFS ≥ 3.09).

**Table 1:** Demographic and clinical characteristics of the whole PD sample, of males and females PD, and of patients PD with and without fatigue

Demographic and clinical variables	PD (*N* = 100)	PD (male)	PD (female)	*p* value	PD-F	PD non-F	*p* value
		(*N* = 59)	(*N* = 41)	PD-M vs. PD-F	(*N* = 36)	(*N* = 64)	PD-fatigue vs. PD-non-fatigue
Age (years)	69 (48; 87)	70 (48; 87)	67.4 (60; 85)	0.2	69 (60; 84)	69.2 (48; 87)	0.85
Sex (M/F)	59/41	–	–	–	13/23	46/18	**0.001**
Education (years)	13 (8; 20)	13 (8;20)	13 (8; 18)	0.06	13 (8;20)	13 (8;20)	0.843
Disease duration (years)	5 (0.5; 34)	5 (0.5; 34)	5 (1; 26)	0.2	5 (0.5; 24)	5 (0.5; 34)	0.78
LEDD (mg)	500 (50; 1185)	500 (50;1185)	501 (50; 1152)	0.93	546.5 (100; 1152)	425 (50; 1185)	0.14
H&Y on (1–1.5.5/2/2.5–3.5-4)	27/42/31	2.10 ± 0.72 (1; 4)	2.14 ± 0.92 (1; 4)	0.7	2.31 ± 0.86 (1–4)	1.98 ± 0.75 (1–4)	0.07
MDS-UPDRS I (score)	8 (0; 35)	7 (0;23)	9 (2; 35)	0.93	12.5 (4; 35)	7 (0; 23)	**< 0.001**
MDS-UPDRS II (score)	8 (1; 40)	8 (3; 21)	9 (1; 40)	0.14	12 (2; 40)	7 (1; 21)	**< 0.001**
MDS-UPDRS III (score)	27 (5; 90)	27 (5; 73)	23 (5; 90)	0.92	31 (10; 90)	25 (5; 73)	0.2
MDS-UPDRS IV (score)	2 (0; 24)	1 (0; 9)	2.5 (0; 24)	0.36	3 (0; 24)	0 (0; 9)	**0.02**
MoCA (score)	26 (18; 30)	26 (18; 30)	27 (18; 30)	0.45	26.5 (18; 30)	26 (18; 30)	0.6
Fatigue according to PFS cut off ≥ 3.09 score (yes/no)	36/64	13/59	23/41	**0.001**	–	–	–
PFS (score)	41.5 (10; 79)	38 (16; 72)	53 (10; 79)	**0.003**	59 (50; 79)	32.5 (10; 49)	**< 0.001**
FSS (score)	36 (6; 63)	32 (9; 63)	41 (6; 58)	**0.04**	51 (29; 63)	27 (6; 54)	**< 0.001**
MFIS total (score)	33 (2; 72)	33 (2; 72)	33 (4; 72)	0.75	45.5 (25; 72)	25.5 (2; 71)	**< 0.001**
MFIS physical (score)	16 (0; 32)	16 (1; 34)	16 (0; 32)	0.51	23 (0; 34)	12.5 (1; 31)	**< 0.001**
MFIS cognitive (score)	14 (0; 32)	14 (0; 32)	15 (1; 32)	0.95	20 (3; 32)	12 (0; 32)	**< 0.001**

Values are medians (minimum; maximum). Categorical variables are reported as frequency. P values refer to Mann Whitney test or Chi square test for categorical variables. Statistical significance was accepted for values of p < 0.05

F=fatigue; PFS= Parkinson’s Fatigue Scale; FSS= Fatigue Severity Scale; H&Y= Hoehn and Yahr scale; LEDD= levodopa equivalent daily dose; MDS-UPDRS= Movement disorders society- Unified Parkinson’s disease rating scale; MFIS=Modified Fatigue Impact Scale; MoCA= Montreal Cognitive Assessment; ON= during medication uptake; PD= Parkinson’s disease

### Comparison between males and females PD sub-groups

Results are reported in Table 1. When the total sample was stratified by sex (59 males and 41 females), females were similar to male participants in terms of age, education, LEDD, disease severity (motor, non-motor and cognitive scales), and disease duration. However, the number of females reporting fatigue according to PFS cut-off ≥ 3.09 was higher than males. Moreover, females exhibited a higher fatigue severity as measured by the FSS and the PFS (Table 1).

### Comparison between the “PD-non-fatigue (PD non-F)” and “PD-fatigue (PD-F)” sub-groups

The comparison between the groups stratified according to the presence or absence of fatigue (“PD-non-fatigue” and “PD-fatigue”) is presented in Table 1. The two groups were similar in terms of age, years of education, LEDD, disease duration, and UPDRS III and MoCA scores. The PD-fatigue sub-group included a higher proportion of females than males (*p* = 0.001), with higher scores on MDS-UPDRS Part I (*p* < 0.001), Part II (*p* < 0.001), and Part IV (*p* = 0.02). Moreover, the PD-fatigue sub-group exhibited significantly higher scores in the PFS, FSS, and MFIS scales (p always < 0.001).

### Comparison between sub-groups stratified according to H&Y stage

Sub-groups’ comparisons stratified by H&Y stage are presented in Table 2. Sex distribution did not differ between groups. As the stages of the disease progress, a worsening of motor, non-motor, and cognitive functions becomes evident, along with an increase in disease duration.

Fatigue was significantly higher in participants with H&Y stage > 2 compared to those with H&Y stage < 2 (MFIS total *p* = 0.001; PFS *p* = 0.004; FSS < 0.001) and H&Y stage = 2 (MFIS total *p* < 0.001; PFS *p* = 0.009; FSS = 0.01). However, no differences were found between H&Y stage < 2 and 2 (MFIS total *p* = 0.88; PFS *p* = 0.87; FSS = 0.26). Both the physical and the cognitive sub-scores of MFIS were significantly higher in H&Y stage > 2 compared to H&Y ≤ 2.

**Table 2:** Demographic and clinical comparisons in PD patients with different H&Y stages

	PD-H&Y < 2 (*N* = 27)	PD-H&Y 2 (*N* = 42)	PD-H&Y > 2 (*N* = 31)	*p* Kruskall Wallis	*p* H&Y < 2 vs. H&Y 2	*p* H&Y < 2 vs. H&Y > 2	*p* H&Y 2 vs. H&Y > 2
AGE	65 (48; 85)	67 (50.99; 77)	73 (55; 87)	**0.001**	0.76	**0.008**	**< 0.001**
SEX (M/F)	16/11	27/15	16/15	0.55	–	–	–
DISEASE DURATION	4 (1; 24)	5 (0.5; 15)	10 (1; 34)	**< 0.001**	0.19	**< 0.001**	**< 0.001**
UPDRS I	6 (1; 21)	7 (0; 23)	12 (4; 35)	**< 0.001**	0.21	**< 0.001**	**0.003**
UPDRS II	7 (2;15)	8.5 (1; 22)	14 (3; 40)	**< 0.001**	**0.03**	**< 0.001**	**0.004**
UPDRS III	15 (5; 47)	26.5 (10; 55)	40 (5; 90)	**< 0.001**	**< 0.001**	**< 0.001**	**< 0.001**
UPDRS IV	0 (0; 9)	1.5 (0; 8)	4 (0; 24)	**0.005**	0.25	**0.003**	**0.02**
MoCA	28 (20; 30)	25.5 (18; 30)	28 (19; 30)	**0.01**	**0.004**	0.52	**0.049**
M-FIS TOT	28 (3; 66)	28.5 (2; 72)	42 (22; 72)	**< 0.001**	0.88	**0.001**	**< 0.001**
MFIS physical	12 (1; 33)	14 (1; 34)	20 (0; 32)	**< 0.001**	0.51	**0.001**	**< 0.001**
MFIS cognitive	12 (1; 29)	11.5 (0; 31)	16 (3; 32)	**0.008**	0.53	**0.03**	**0.002**
PFS	33 (16; 72)	40 (10; 69)	49 (26; 79)	**0.008**	0.87	**0.004**	**0.009**
FSS	29 (9; 63)	35.5 (6; 58)	41 (14; 57)	**0.001**	0.26	**< 0.001**	**0.01**

Values are medians (minimum; maximum). Categorical variables are reported as frequency. P values refer to Kruskall Wallis test and Mann Whitney test. Statistical significance was accepted for values of p < 0.05

F=female; M=male; PFS= Parkinson’s Fatigue Scale; FSS= Fatigue Severity Scale; H&Y= Hoehn and Yahr scale; MDS-UPDRS= Movement disorders society- Unified Parkinson’s disease rating scale; MFIS=Modified Fatigue Impact Scale; MoCA= Montreal Cognitive Assessment; ON= during medication uptake; PD= Parkinson’s disease

### Spearman’s correlational analyses

#### Whole sample

**Fig. 1:**
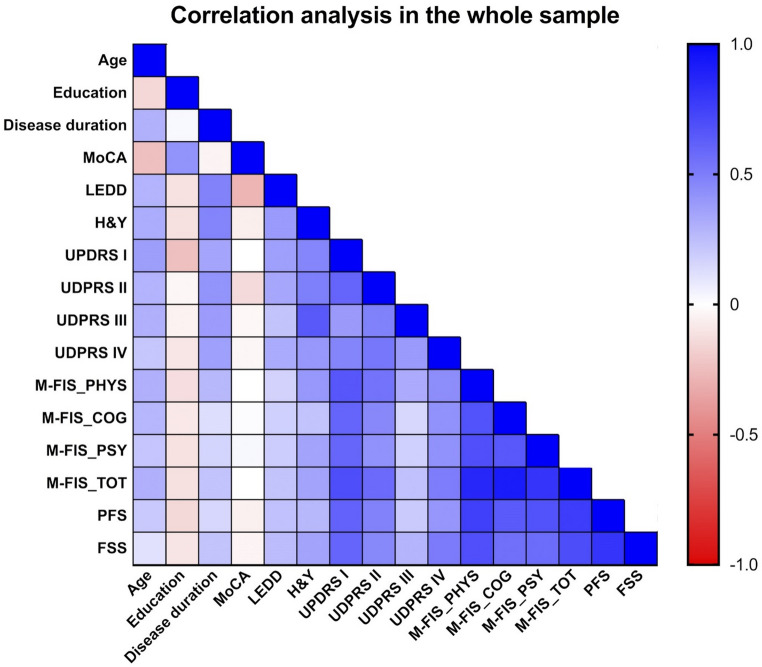
Results of correlation analyses in the whole group of patients with Parkinson’s disease (heatmap). Colors denote Spearman’s correlation coefficient

The results of correlation analyses are depicted in Fig. 1. PFS, FSS, and MFIS demonstrated strong significant intercorrelations, with correlation coefficients ranged from 0.701 to 0.806, *p* < 0.001. The higher scores on one scale were associated with higher scores on the others.

Both cognitive and physical dimensions of fatigue showed weak but significant correlation with age (MFIS total ρ = 0.302 *p* = 0.002; MFIS physical ρ = 0.299 *p* = 0.002; MFIS cognitive ρ = 0.282 *p* = 0.004; PFS ρ = 0.204 *p* = 0.042), with disease duration (MFIS total ρ = 0.230 *p* = 0.021; MFIS physical ρ = 0.274 *p* = 0.006; FSS ρ = 0.225 *p* = 0.024), and with LEDD (MFIS total ρ = 0.224 *p* = 0.028; PFS ρ = 0.236 *p* = 0.020; FSS ρ = 0.254 *p* = 0.012).

Positive correlations were also found between Fatigue scales (MFIS, PFS, FSS) and MDS-UPDRS part I, II, III, IV scores, indicating that more severe motor and non-motor symptoms were associated with a greater subjective experience of fatigue. Specifically, moderate to strong positive correlations were found between:

- *MDS-UPDRS I (Non-Motor Aspects of Experiences of Daily Living)) with*: MFIS total (ρ = 0.698 *p* < 0.001), MFIS physical sub-score (ρ = 0.661 *p* < 0.001), and cognitive sub-score (ρ = 0.603 *p* < 0.001); PFS (ρ = 0.608 *p* < 0.001); FSS (ρ = 0.601 *p* < 0.001), thus indicating that higher levels of fatigue are associated with more pronounced non-motor symptoms.
- *MDS-UPDRS II (Motor Aspects of Experiences of Daily Living)) with*: MFIS total (ρ = 0.573 *p* < 0.001), MFIS physical sub-score (ρ = 0.548 *p* < 0.001), and cognitive sub-score (ρ = 0.461 *p* < 0.001); PFS (ρ = 0.485 *p* < 0.001); FSS (ρ = 0.465 *p* < 0.001), suggesting that patients with greater fatigue experience more difficulties in daily activities.
- *MDS-UPDRS IV (Motor Complications) with*: MFIS total (ρ = 0.502 *p* < 0.001), MFIS physical sub-score (ρ = 0.437 *p* < 0.001), and cognitive sub-score (ρ = 0.421 *p* < 0.001); PFS (ρ = 0.406 *p* < 0.001); FSS (ρ = 0.512 *p* < 0.001), suggesting that motor complications, such as dyskinesia and fluctuations, may contribute to fatigue.

Finally, weak correlations were found between *MDS-UPDRS III (Motor Examination)* and: MFIS total (ρ = 0.225 *p* = 0.024), MFIS physical sub-score (ρ = 0.241 *p* = 0.016); FSS (ρ = 0.263 *p* = 0.008).

### PD-fatigue group

**Fig. 2:**
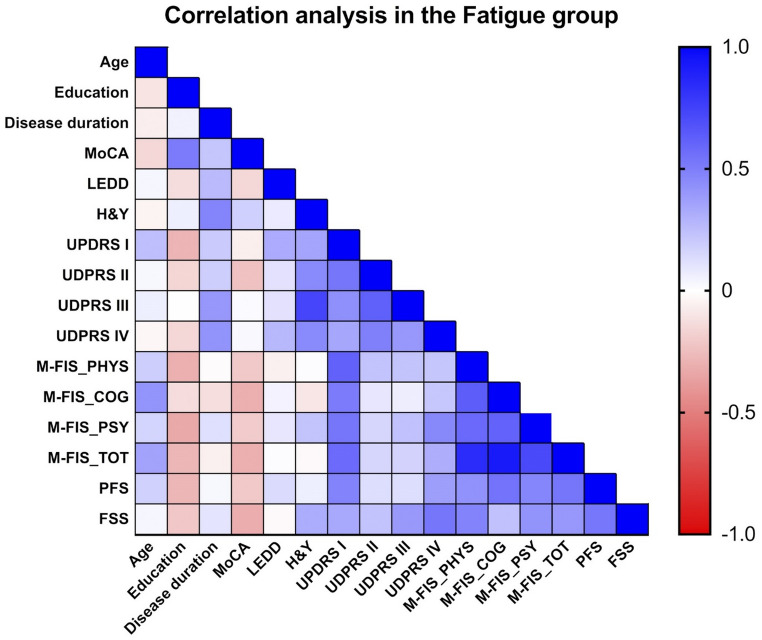
Results of correlation analyses in the group of Parkinson’s disease patients with fatigue (heatmap). Colors denote Spearman’s correlation coefficient

The results of correlation analyses are depicted in Fig. 2. Results confirmed a moderate association between age and fatigue (specifically in the cognitive component) as shown by MFIS total (ρ = 0.356 *p* = 0.033) and MFIS cognitive sub-score (ρ = 0.391 *p* = 0.019). However, no association was found between fatigue and disease duration.

The PD-fatigue group showed strong to moderate positive correlation with non-motor symptoms, particularly between *MDS-UPDRS I (Non-Motor Aspects of Experiences of Daily Living)* and MFIS total (ρ = 0.573 *p* = 0.001), MFIS physical (ρ = 0.615 *p* < 0.001) and cognitive (ρ = 0.511 *p* = 0.003) sub-scores; PFS (ρ = 0.482 *p* = 0.005) and FSS (ρ = 0.332 *p* = 0.063^**§**^) total scores.

Additionally, *MDS-UPDRS III (Motor Examination)* showed a moderate correlation with fatigue perception as expressed by FSS (ρ = 0.395 *p* = 0.017) and *MDS-UPDRS IV (Motor Complications)* showed moderate correlation with fatigue symptom as measured by PFS (ρ = 0.374 *p* = 0.045) and FSS (ρ = 0.532 *p* = 0.003). No correlation was found between fatigue and *MDS-UPDRS II (Motor Aspects of Experiences of Daily Living)*.

Finally, in this sample, there was a trend toward a negative association between global cognitive status and fatigue. Specifically, MoCA score was associated with MFIS total (ρ=−0.302 *p* = 0.073^**§**^), cognitive sub-score (ρ=−0.305 *p* = 0.071^**§**^); and FSS (ρ=−0.318 *p* = 0.059^**§**^).

## Discussion

Fatigue is a highly prevalent and debilitating non-motor symptom in PD, with significant implications for quality of life and functional independence [25]. This study aimed to investigate the prevalence of fatigue and its associations with sociodemographic factors, disease severity, and motor, non-motor, and cognitive symptoms in an Italian cohort of patients with PD.

In our sample (n.100 participants), fatigue was present in 36% of individuals with PD, consistent with existing literature identifying fatigue as a common symptom in PD, affecting 33–58% of patients [4, 36]. Overall, our results revealed that patients with fatigue exhibited more severe non-motor symptoms, particularly mood disturbances, sleep dysfunctions, and autonomic issues, as well as disease complications such as dyskinesia and fluctuations, highlighting its biopsychosocial underpinnings. Moreover, PD patients with fatigue showed higher impact on experiences of daily living (UPDRS II) as previously reported [36], despite having a similar severity of motor and cognitive features compared to subjects without fatigue.

To substantiate the aforementioned hypothesis, fatigue demonstrated strong correlations with non-motor aspects of PD, particularly as measured by the MDS-UPDRS Part I. For example, MFIS total and sub-scale scores (physical and cognitive) strongly correlated with MDS-UPDRS I scores, revealing the role of non-motor symptoms, such as mood disturbances, sleep dysfunctions, and autonomic issues, in exacerbating the subjective experience of fatigue [25].

In contrast, correlations between fatigue and motor symptoms (MDS-UPDRS III) were relatively weak, indicating that motor symptoms may contribute only partially to fatigue compared to the stronger associations observed for non-motor features. Similarly, motor complications (MDS-UPDRS IV) showed moderate correlations with fatigue scales, especially with FSS and PFS scores, likely reflecting the impact of dyskinesias and motor fluctuations on physical and psychological energy reserves [37]. However, it is important to note that we also observed a moderate correlation between UPDRS I and IV, suggesting that motor complications and non-motor symptoms may be interrelated, potentially contributing together to fatigue in a multivariate context.

A significant gender difference was also observed, with female participants reporting more frequent and higher fatigue levels than males. This finding aligns with prior research, suggesting that women with PD experience more severe non-motor symptoms, including fatigue, depression, and pain [5–7, 36]. Interestingly, these differences were not mirrored in other measures of disease severity such as motor and cognitive scores, suggesting that gender-specific biological or psychosocial factors may influence the perception or reporting of fatigue.

The relationship between fatigue and disease severity was evident across several dimensions. Fatigue scores (PFS, FSS, MFIS) were significantly higher in patients with more advanced stages of the disease (H&Y > 2), supporting previous studies that link fatigue with disease progression [8]. This was particularly evident when comparing H&Y stages 2 and 3, while no significant differences were observed between earlier stages (H&Y < 2 and 2). The increase in both the physical and cognitive MFIS sub-scores in later stages highlights the growing burden of fatigue as both a physical and cognitive phenomenon in PD [11, 12, 38]. In our sample, in the early phases (H&Y < 2), motor symptoms were primarily observed, while non-motor symptoms (assessed by the MDS-UPDRS-I) and fatigue scores were lower.

Interestingly, fatigue was weakly but significantly correlated with age in the overall sample, whereas no such association was observed in the subgroup of individuals with fatigue. This may suggest a general, modest influence of age on fatigue perception, potentially related to age-associated physiological changes, although this effect appears less pronounced among individuals already experiencing fatigue. Similarly, weak correlations between fatigue and disease duration indicate that longer disease duration is not necessarily associated with greater fatigue, highlighting that multiple factors likely contribute to fatigue severity throughout the disease course.

The distribution of individuals across H&Y stages in both the fatigue and non-fatigue groups further supports the notion that fatigue is not merely an expression of disease severity [25]. Instead, it may arise from the dysfunction of non-dopaminergic pathways that develop as the disease progresses [3, 25]. Neuroimaging studies employing [123I]ß-CIT SPECT [39] and 18 F-Dopa PET scans [24] have not identified a clear correlation between fatigue and nigrostriatal denervation. In our sample, higher levels of fatigue correlated with higher LEDD, supporting the hypothesis that fatigue is only partially sensitive to dopaminergic treatment [27–29]. Recent evidence [40] suggests that dopaminergic mechanisms may contribute to fatigue perception in the early disease stages, while other neurotransmitter systems may become more relevant as the disease progresses. Notably, serotonergic denervation in basal ganglia and limbic circuits was observed in fatigued PD patients [24], and fatigue severity was negatively correlated with serotonin levels in cerebral spinal fluid (CSF) of PD patients [41]. Serotonergic dysfunction is associated to PD-related fatigue, depression, and anxiety, indicating shared neurobiological mechanisms [42, 43]. These neurotransmitter imbalances in basal ganglia and limbic circuits may disrupt the integration of sensory, motor, and affective functions [24]. Moreover, glutamatergic overactivity and the consequent neuroinflammation have been recently considered key mechanisms underlying primary fatigue [13, 20, 37].

The observed trend toward a negative association between cognitive status (as measured by MoCA scores) and fatigue, although not statistically significant, is noteworthy. Lower cognitive performance may contribute to heightened perception of fatigue, particularly in its cognitive dimensions [11, 29]. Experimental evidence [44] suggests that beyond the sensorimotor and limbic loops of the cortico-basal ganglia network, areas involved in attention, executive functions, and memory may also be significantly affected in patients experiencing fatigue. However, larger studies are needed to clarify this relationship.

The findings of this study have several clinical implications. First, the high prevalence of fatigue and its strong associations with non-motor symptoms highlight the need for systematic screening in PD patients [12, 25, 45]. The significant intercorrelations among the PFS, FSS, and MFIS scores underscore the robustness of these instruments in capturing the subjective experience of fatigue, suggesting that these scales measure overlapping dimensions. These tools should be integrated into routine clinical evaluations to better assess the multidimensional nature of fatigue [33].

Second, the observed gender differences suggest the need for tailored interventions in female patients, including psychosocial support, gender-sensitive counseling, and targeted pharmacological approaches [7].

Third, the correlations between fatigue and non-motor symptoms underscore the importance of addressing comorbidities such as depression, sleep disturbances, and autonomic dysfunction. Different interventions such as cognitive-behavioral therapy, physical activity programs, and pharmacological treatments, may also help alleviate fatigue [12, 27–29, 46].

This study has several limitations that warrant consideration. The cross-sectional design limits the ability to infer causal relationships between fatigue and other clinical variables. Longitudinal studies are needed to explore how fatigue evolves over disease progression and in response to therapeutic interventions. No formal sample size calculation was performed to ensure representativeness of the Italian PD population. Larger cohorts representative of the entire national territory are necessary to validate these findings and explore sub-groups differences, particularly in less common PD phenotypes. Another potential limitation is the absence of a specific questionnaire to assess non-motor symptoms. We selected the UPDRS I for its practicality, ease of use in settings like waiting rooms, and its effectiveness in evaluating a range of symptoms which are sufficient for our study. However, future studies should consider including more comprehensive evaluations of cognitive, behavioral, and sleep changes. In this context, while bivariate Spearman correlations were appropriate for the exploratory nature of the present study, this approach does not account for potential confounding variables, nor does it capture more complex multivariate relationships among the variables. Future research could benefit from the use of multivariate statistical models that allow simultaneous adjustment for multiple factors. In particular, longitudinal studies would be valuable to investigate factors that predict the development of fatigue over time and to better clarify the underlying relationships. Additionally, examining the biological mechanisms underlying fatigue in PD, such as neuroinflammation, mitochondrial dysfunction, and neurotransmitter imbalances will be crucial. Finally, research on the impact of specific treatments (both pharmacological and rehabilitative) on fatigue levels will be essential to develop effective management strategies.

This study highlights the complex and multifactorial nature of fatigue in PD patients. Fatigue is closely linked to non-motor symptoms, disease severity, and gender, underscoring the need for comprehensive and personalized approaches to its assessment and management. Addressing fatigue requires a holistic understanding of its physical, cognitive, and emotional dimensions, as well as targeted interventions which address both motor and non-motor aspects of the disease. Through such efforts, it may be possible to improve the quality of life for individuals living with PD and their caregivers.

## Data Availability

The dataset used and analyzed during the current study is available from the corresponding author upon request to qualified researchers (i.e., affiliated to a university or research institution/hospital).
